# Löfgren's syndrome manifestation of acute sarcoidosis: short-term resolution with association of anti-inflammatory drugs^⋆^

**DOI:** 10.1016/j.abd.2023.04.011

**Published:** 2024-04-23

**Authors:** Rebecca Perez de Amorim, Ana Flávia Teixeira de Abreu, Aline Garcia Lutz, Vinícius Cardoso Nóbrega, Ivanka Miranda de Castro, Hélio Amante Miot

**Affiliations:** Department of Infectious Diseases, Dermatology, Imaging Diagnosis and Radiotherapy, Faculty of Medicine, Universidade Estadual Paulista, Botucatu, SP, Brazil

*Dear Editor,*

A 43-year-old woman, with no previous comorbidities, came to the outpatient clinic after being referred by the Infectious Diseases Division. Twenty-one days before, she had developed a painful lesion on her left calf, dry cough and persistent fever (38 °C). After five days, lesions characterized as violet nodules appeared on the legs (Figure 1) and upper limbs (Figure 2). A thorough physical examination was performed, with palpation of the joints of the hands, wrists, elbows, knees, and ankles. There was joint edema and erythema on the knees and left metacarpophalangeal joints. She denied previous use of medication.

**Figure 1:**
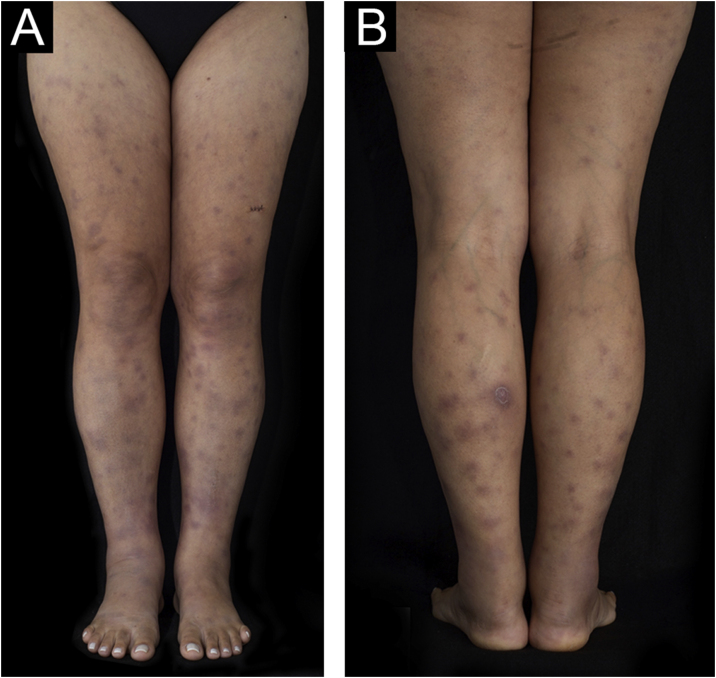
Multiple erythematous and edematous nodules on the lower limbs. (A) Anterior view. (B) Posterior view.

**Figure 2:**
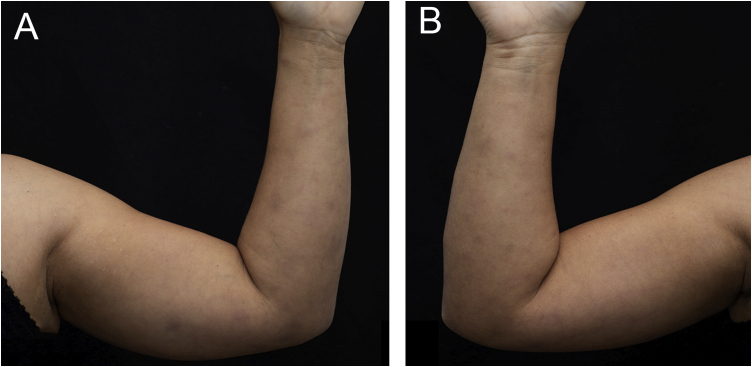
Multiple erythematous and edematous nodules on the upper limbs. (A) Medial aspect of the left upper limb. (B) Medial aspect of the right upper limb.

A chest tomography was requested (Figure 3), which showed perihilar lymph node disease and peripheral centrilobular micronodules. Recent serology for syphilis, HIV, hepatitis B and C, and blood cultures were negative. There was an increase in erythrocyte sedimentation rate (66 mm/h) and C-reactive protein level (8.8 mg/dL). The tuberculin skin test (PPD) and antinuclear factor were negative. The 24-h urinary calcium and angiotensin-converting enzyme levels were unchanged. Subcutaneous adipose tissue histopathology showed changes compatible with erythema nodosum – septal lymphocytic panniculitis, without vasculitis, with granuloma formation and presence of multinucleated giant cells (Figure 4). The patient ocular fundus examination showed no alterations. No adeno/visceromegaly was identified on clinical examination.

**Figure 3:**
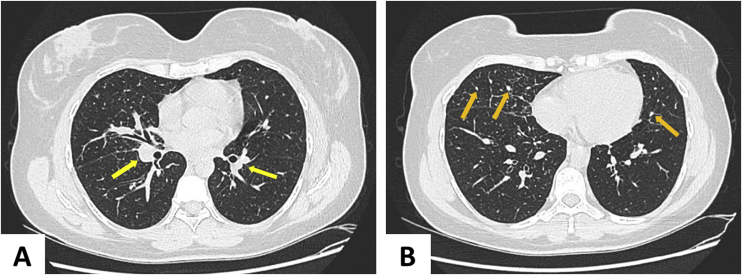
Computed tomography of the chest showing parabronchial nodules – yellow arrows (A) and multiple peripheral centrilobular micronodules – orange arrows (B).

**Figure 4:**
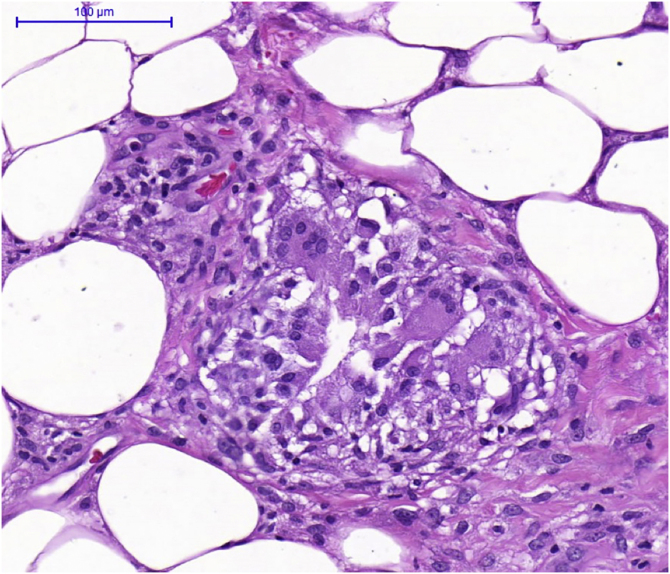
Subcutaneos adipose tissue histopathology showing septal lymphocytic panniculitis, without vasculitis, granuloma formation and presence of multinucleated giant cells.

The signs and symptoms allowed the diagnosis of Löfgren’s syndrome (LS), considered an acute manifestation of sarcoidosis and occurring in 5% to 10% of cases of the disease.^1^, ^2^ The patient was treated with prednisone 60 mg/day, hydroxychloroquine 400 mg/day, methotrexate 15 mg/week, and folic acid 5 mg/week. After five days, she no longer had a fever, and the pain and lesions had improved. After 30 days, she had a complete resolution of the complaints and the erythema nodosum. The corticosteroid was completely discontinued in 90 days, the antimalarial in 120 days, and methotrexate in 12 months. The control CT carried out ten months later, showed minimal micronodular findings, leading to discharge from the Pulmonology Division. At the 15-month follow-up, the patient remains asymptomatic.

LS mainly affects people between 25 and 30 to 40 years old, with a similar incidence between genders. There is, however, a second peak between 45 and 65 years of age, when 70% of cases are in women.^1^, ^2^ The presence of the syndrome suggests a good prognosis, with 85% of patients showing spontaneous resolution of the condition within two years of symptom onset. The classic triad of symptoms includes erythema nodosum (60%), bilateral hilar lymphadenopathy (100%), and arthritis/arthralgias (10% to 30%). The diagnosis requires two of the three cardinal symptoms – when all are present the specificity of the clinical diagnosis is 95%.^2^, ^3^, ^4^

Most patients with LS do not have respiratory symptoms; nevertheless, parenchymal radiographic changes are observed in 90% of the cases. Stage I disease (hilar adenopathy without pulmonary infiltrates) resolves in 60% to 80% of the patients.^3^, ^4^

A biopsy of the affected organs should be performed to exclude infection, rheumatological disease, or malignancy. The best location depends on the accessibility, safety and feasibility of the procedure. Biopsies of superficial lesions – non-erythema nodosum skin lesions or palpable peripheral lymph nodes – are preferable. The pathognomonic finding of sarcoidosis is a well-formed, noncaseating epithelioid granuloma. Erythema nodosum-like lesions present as septal panniculitis that may have a granulomatous outline (Fig. 4).^5^

PPD is necessary as a screening tool for tuberculosis. The test is negative in patients with sarcoidosis; when positive, the diagnosis of tuberculosis should be considered.^5^, ^6^, ^7^

Treatment is symptomatic, in most cases with non-steroidal anti-inflammatory drugs and rest. Glucocorticoids are associated with clinical and pulmonary function improvement, without changing the course of the disease. The use of chloroquine has been reported in patients with cutaneous sarcoidosis, hypercalcemia, hypercalciuria and in neurosarcoidosis refractory to corticosteroids. Methotrexate administered at a dose between 7.5-15 mg/week is well tolerated and accepted in cases of lung, muscle and skin changes.^2^, ^5^, ^6^, ^7^

## Financial support

None declared.

## Authors' contributions

Rebecca Perez de Amorim: Design and planning of the study; drafting and editing of the manuscript; collection, analysis and interpretation of data; effective participation in research orientation; intellectual participation in the propaedeutic and/or therapeutic conduct of the studied cases; critical review of the literature; critical review of the manuscript; approval of the final version of the manuscript.

Ana Flávia Teixeira de Abreu: Drafting and editing of the manuscript; collection, analysis and interpretation of data; intellectual participation in the propaedeutic and/or therapeutic conduct of the studied cases; critical review of the literature; critical review of the manuscript.

Aline Lutz Garcia: Collection, analysis and interpretation of data.

Vinícius Cardoso Nóbrega: Collection, analysis and interpretation of data.

Ivanka Miranda de Castro: collection, analysis and interpretation of data; intellectual participation in the propaedeutic and/or therapeutic conduct of the studied cases.

Hélio Amante Miot: Design and planning of the study; drafting and editing of the manuscript; effective participation in research orientation; intellectual participation in the propaedeutic and/or therapeutic conduct of the studied cases; critical review of the literature; critical review of the manuscript; approval of the final version of the manuscript.

## Conflicts of interest

None declared.
